# Meta-learner-based frameworks for interpretable email spam detection

**DOI:** 10.3389/frai.2025.1569804

**Published:** 2025-10-21

**Authors:** Meghana Kshirsagar, Vedant Rathi, Conor Ryan

**Affiliations:** ^1^Biocomputing Developmental Systems Research Group, Department of Computer Science and Information Systems, University of Limerick, Limerick, Ireland; ^2^Lero, Research Ireland Research Centre for Software, Limerick, Ireland; ^3^University of Illinois Urbana-Champaign, Champaign, IL, United States

**Keywords:** machine learning, deep learning, spam email detection, natural language processing, classification, meta-learner, algorithmic bias, data bias

## Abstract

**Introduction:**

With the increasing reliance on digital communication, email has become an essential tool for personal and professional correspondence. However, despite its numerous benefits, digital communication faces significant challenges, particularly the prevalence of spam emails. Effective spam email classification systems are crucial to mitigate these issues by automatically identifying and filtering out unwanted messages, enhancing the efficiency of email communication.

**Methods:**

We compare five traditional machine-learning and five deep-learning spam classifiers against a novel meta-learner, evaluating how different word embeddings, vectorization schemes, and model architectures affect performance on the Enron-Spam and TREC 2007 datasets. The primary aim is to show how the meta-learner's combined predictions stack up against individual ML and DL approaches.

**Results:**

Our meta-learner outperforms all state-of-the-art models, achieving an accuracy of 0.9905 and an AUC score of 0.9991 on a hybrid dataset that combines Enron-Spam and TREC 2007. To the best of our knowledge, our model also surpasses the only other meta-learning-based spam detection model reported in recent literature, with higher accuracy, better generalization from a significantly larger dataset, and lower computational complexity. We also evaluated our meta-learner in a zero-shot setting on an unseen real-world dataset, achieving a spam sensitivity rate of 0.8970 and an AUC score of 0.7605.

**Discussion:**

These results demonstrate that meta-learning can yield more robust, bias-resistant spam filters suited for real-world deployment. By combining complementary model strengths, the meta-learner also offers improved resilience against evolving spam tactics.

## 1 Introduction

The growing popularity of email in the modern era has been fueled by an increase in internet usage and its universality, connecting people across the world, and reliability, permitting the distribution of ideas swiftly (International Telecommunication Union, 2023). According to one report, daily email traffic has grown by more than 4% yearly and the number of users by 3%. Current projections estimate that there will be at least 4.5 billion email users in 2025 (The Radicati Group, 2021).

However, with the rise of rapid and convenient communication comes significant risks. One of the main risks has been the growth of spam emails, unsolicited emails sent in bulk, which can take various forms, including phishing, malware distribution, frauds, and commercial advertising (Ghourabi and Alohaly, 2023). Spam (phishing) emails can be especially dangerous if they lead people to share private information like credit card numbers (Butt et al., 2023) or viruses that can hack into desktops and steal sensitive data (Ayo et al., 2024). In particular, spear phishing poses a heightened threat, as these targeted attacks use personalized messages to deceive individuals into divulging sensitive information. Often, these emails are crafted to look like they come from someone the recipient knows personally (Badman, 2023). These dangers can result in significant financial losses to individuals and organizations. One study estimates that Americans suffer costs of approximately $20 billion per year because of spam, while spammers collect global revenues of about $200 million yearly (Rao and Reiley, 2012).

Costs incurred due to spam emails will continue to grow as the traffic of emails sent increases yearly. Spam emails sent daily have increased from 2.4 billion in 2002 to 158.4 billion in 2023 (Almeida and Yamakami, 2012; Statista, 2024), reflecting a remarkable geometric growth rate of 22.1% annually. Moreover, while general spam emails are not inherently directed toward any specific race or gender, the content of such emails often caters to working-age males, which may influence the feelings people of different demographics have toward these emails as the content may be less relevant to certain groups (Grimes et al., 2007). Nonetheless, certain spam, such as phishing, may be especially targeted toward senior individuals due to their perceived vulnerability stemming from potential unfamiliarity with the technology and their higher potential for having larger wealth (Lin et al., 2019).

Consequently, spam detection techniques have gained significant traction, as spammers employ increasingly sophisticated methods to limit people's abilities to distinguish genuine content (termed ham) from spam (Ayo et al., 2024). Most of these spam detection techniques can be categorized as either machine learning-based or deep learning-based. Of machine learning-based methods, frequently used models include Random Forest (Abkenar et al., 2021; Vinitha and Renuka, 2019; Ageng et al., 2024), Support Vector Machine (Khamis et al., 2020; Ma et al., 2020), Naive Bayes (Ma et al., 2020; Zraqou et al., 2023), XGBoost (Omotehinwa and Oyewola, 2023; Dhar et al., 2023), and more. Common deep-learning based approaches include using individual models or a combination of the following: CNN (Yang et al., 2019; Metlapalli et al., 2020; Rahman and Ullah, 2020), LSTM (Yang et al., 2019; Metlapalli et al., 2020; Basyar et al., 2020; Poomka et al., 2019), GRU (Basyar et al., 2020; Poomka et al., 2019; Wanda, 2023), BiLSTM (Rahman and Ullah, 2020; Abayomi-Alli et al., 2022), etc.

Despite these recent advancements in spam detection techniques, substantial challenges persist, limiting the interpretability and generalizability of these models (Gilpin et al., 2018). In particular, many existing approaches suffer drawbacks related to data or algorithmic biases. Data bias refers to data that is limited in certain ways, preventing the sampled data from accurately portraying population trends. For example, spam-detecting models trained on only one dataset (Lee et al., 2010; Faris et al., 2017) often fail to capture spam characteristics across diverse sources, limiting the model's ability to be applied in real-world contexts. Thus, data biases can often cause algorithmic bias, which refers to systematic errors in computer systems resulting in unfair (biased) outcomes (Kordzadeh and Ghasemaghaei, 2022). For instance, spam-detecting models trained with only one source may fail to detect spam emails with varying contexts, thereby exhibiting a bias toward certain types of emails over others.

To contribute to growing research and address existing issues in spam-filtering techniques, in this study, we conduct a comprehensive evaluation of both machine learning and deep learning-based spam detection techniques. We compare five different ML models (Random Forest, Support Vector Machine, Naive Bayes, XGBoost, Logistic Regression) and five DL models (LSTM, BiLSTM, GRU, BiGRU, CNN). We compare two feature-selection algorithms for ML-based techniques, TF-IDF vectorization and Bag of Words vectorization. On the deep learning side, we compare two types of word embedding algorithms, GloVe and Word2Vec, and two types of architectures, models with attention vs. models without attention. Finally, we propose a meta-learner model that combines the predictions of all ML models and outperforms state-of-the-art models. We instantiate these pipelines on three datasets: Enron-Spam, TREC 2007, and a hybrid dataset combining the former two. The meta-learner is also tested on an unseen real-world dataset. Our primary research contributions are:

- Conducting a comprehensive evaluation of different spam detection algorithms;
- Comparing model performances with different feature-selection approaches, word embeddings, and architectures;
- Evaluating results on a hybrid dataset combining multiple constituent datasets to mitigate data bias;
- Proposing a new meta-learner model and comparing it with state-of-the-art models on key performance metrics;
- Testing in a zero-shot setting to evaluate the generalizability and robustness of the spam detection models.

The rest of the paper is structured as follows: Section 2 summarizes related literature (2.1) which also develops email spam detection techniques, the mathematical bases (2.2) of the algorithms employed in our research, the data methodology (2.3), and our experimental setup (2.4). Section 3 covers our study results, and finally, Section 4 explores the real-world relevance of our research and promising future steps.

## 2 Materials and methods

### 2.1 Related work

This section delves into the related literature concerning spam classification in machine learning and deep learning methodologies. We aim to identify trends, gaps, and diverse approaches that inform our research. The selected papers were chosen based on their relevance to the scope of our study, the datasets they evaluated, and/or the classification approaches they employed. Specifically, we focused on works that address email spam classification, as this is the primary focus of our research. By including studies that utilize benchmark datasets such as Enron-Spam, TREC 2007, and SpamAssassin, we ensure a fair and consistent comparison with our proposed methods. Additionally, we prioritized papers that explore a variety of techniques, including traditional machine learning models, deep learning architectures, hybrid approaches, and novel methodologies, as well as ensemble methods, feature engineer techniques, and transformer-based models, to provide a comprehensive overview of the field. Metrics of the following models which used Enron-Spam or TREC 2007 datasets are omitted in this section as their performances are compared in the results section, Subsection 3.4. To provide a structured and comprehensive overview of the existing literature, we organize the related works into six primary categories based on their methodologies and contributions.

#### 2.1.1 Hybrid and attention-based models

Zavrak and Yilmaz (2023) proposed a hybrid spam classification model using CNNs, bidirectional GRUs, and attention mechanisms. Attention at word and sentence levels captured semantic relationships, with two architectures tested: a standard CNN and a temporal convolutional network (TCN). Evaluated on TREC 2007, GenSpam, SpamAssassin, Enron-Spam, and LingSpam, experiments included (a) same-dataset training/testing (five experiments) and (b) cross-dataset evaluation, training on one dataset and testing on the other four (20 experiments).

Krishnamoorthy et al. (2024) developed a hybrid DNN-BiLSTM model for Enron-Spam. Using TF-IDF features and handcrafted metrics like word counts and capitalization, the model trained with triple cross-validation and outperformed multiple ML and DL baselines across all metrics.

Alsudani et al. (2024) used crow search optimization (CSO) to tune weights in a hybrid feedforward neural network-LSTM. Inspired by crows' food-storing behavior, CSO improved learning efficiency, achieving strong results on the SpamAssassin Public Corpus.

#### 2.1.2 Transformer-based and pre-trained language models

Guo et al. (2023) used BERT embeddings with traditional classifiers for spam detection. Embeddings from a pre-trained BERT model on Enron-Spam and Spam or Not Spam were used with SVM, KNN, Random Forest, and Logistic Regression, with Logistic Regression achieving the highest AUC, F-score, and precision.

Tida and Hsu (2022) proposed a universal spam detection model based on BERT Base with 12 encoders and 110M parameters. Pre-trained weights reduced computation, and additional linear, dropout, and ReLU layers were added. Tested on Enron-Spam, SpamAssassin, LingSpam, and SpamText, extensive tuning of batch sizes and splits showed best results with a batch size of 128.

Uddin and Sarker (2024) applied finetuned DistilBERT, a lightweight BERT variant, for phishing email classification. Using 20,000 emails with oversampling to handle imbalance, they tested various batch sizes, learning rates, and epochs. LIME and Transformer Interpret improved explainability by highlighting token-level contributions in predictions.

#### 2.1.3 Feature engineering and dimensionality reduction

Wang et al. (2021) introduced a manifold learning-based approach to improve spam detection efficiency. Using the Laplacian score to select key features and applying the LEP algorithm for dimensionality reduction, they improved SVM accuracy and reduced computation. On Enron-Spam, GenSpam, and PU1 (70–30 split), performance matched baselines but with faster processing.

Chu et al. (2020) proposed a two-phase spam detection framework using the C4.5 decision tree. Email headers were analyzed first, then bodies if uncertain, using keyword tables for spam and non-spam mapping. Evaluated on TREC 2007 and Enron-Spam subsets with a 50–50 split, false positives decreased, though false negatives slightly increased.

Ayo et al. (2024) designed a hybrid deep learning model with a fuzzy inference system. Genetic search and CfsSubsetEval optimized feature selection, while fuzzy logic subdivided spam severity to reduce misclassification. On UCI SpamBase, the model achieved high true positive rates and low processing time.

#### 2.1.4 Ensemble and meta-learning approaches

Adnan et al. (2024) proposed a meta-classifier combining five models—Logistic Regression, Decision Tree, KNN, Gaussian Naive Bayes, and AdaBoost. Evaluated on a combined Enron-Spam and SpamAssassin dataset with Logistic Regression as the meta-classifier, their ensemble outperformed all individual models across F-score, precision, and other metrics.

Omotehinwa and Oyewola (2023) explored hyperparameter tuning for Random Forest and XGBoost. Using grid-search with 10-fold cross-validation on the Enron dataset, tuned models outperformed baselines, with XGBoost achieving the highest overall accuracy.

Fatima et al. (2024) developed an optimized spam detection framework using CountVectorizer, TF-IDF, and multiple models including Naive Bayes, Extra Trees, XGBoost, Random Forest, MLP, and SGD. Tested on Ling Spam, UCI SMS Spam, and a new dataset, SGD performed best, and combining count-based vectorization with hyperparameter tuning further improved accuracy.

#### 2.1.5 Novel methodologies in spam detection

Ezpeleta et al. (2020) integrated sentiment analysis and personality detection into spam datasets. Sentiment was derived from dictionaries, and personality traits from the Myers-Briggs model. Testing 10 Bayesian spam filters on TREC 2007 and CSDMC2010 showed combining both features improved accuracy over using either alone.

Lee et al. (2023) developed BlindFilter, a privacy-preserving framework combining homomorphic encryption with Naive Bayes. Encryption enabled computation on protected data, with WordPiece tokenization used for representation. BlindFilter's four stages—key generation, encryption, classification, and decryption—were evaluated on Enron-Spam and TREC 2007, achieving best results on TREC 2007 with a 20–20–60 split.

Mythili et al. (2024) extended Multinomial Naive Bayes by introducing a temporal classifier. Standard preprocessing (tokenization, stemming, lemmatization, stop-word removal) was used, while temporal features such as arrival times, sender frequency, and keyword distributions were added. Incorporating temporal dependencies improved accuracy on the “Spam filter” dataset.

#### 2.1.6 Traditional machine learning and deep learning models

Ghogare et al. (2023) compared Naive Bayes, SVM, and Random Forest on datasets with four preprocessing strategies: standard, lemmatization, stemming, and none. Using Enron-Spam and SpamAssassin, preprocessing effects varied by model, and their classifier outperformed Yahoo's built-in spam filter.

Chirra et al. (2020) evaluated deep learning models for binary and multi-class spam classification. On Enron, CNN, LSTM, and GRU were tested for binary tasks, while RNN, LSTM, GRU, BiRNN, BiLSTM, and BiGRU were applied to the 20 Newsgroup dataset. GloVe embeddings captured semantics, and dropout mitigated overfitting. CNN performed best on mini-Enron, while RNN and BiRNN tied on 20 Newsgroup.

Rabbi et al. (2023) compared Logistic Regression, KNN, AdaBoost, Multinomial Naive Bayes, Gradient Boosting, and Random Forest on LingSpam and TREC 2007. Using TF-IDF and standard preprocessing, AdaBoost performed best on LingSpam, Random Forest on TREC 2007. KNN, Naive Bayes, and Logistic Regression trained fastest.

### 2.2 Mathematical formulations

We first conduct a thorough comparison of machine learning and deep learning models using a pipelined approach on three datasets—Enron-Spam, TREC 2007, and a hybrid dataset—and then combine their results. More details on the datasets can be found in Section 2.3.

For machine learning models, we compare two vectorization approaches with five different models. The approaches are term frequency-inverse document frequency and bag of words, and the models are XGBoost, Random Forest, Logistic Regression, Naive Bayes, and Support Vector Machine. For deep learning models, we compare two types of word embeddings and two types of model architectures with five different models. The word embeddings used are GloVe and Word2Vec. The deep learning architectures we explore and models with attention layers and models without attention layers. The models compared are LSTM, BiLSTM, GRU, BiGRU, and CNN. Then, we propose a machine learning-based meta-learner model that outperforms all other models; it is tested in a zero-shot setting on an unseen real-world dataset.

#### 2.2.1 Meta-learner system overview

##### 2.2.1.1 Meta-learner

Meta-learning refers to algorithms that learn from the outputs of other learning models to optimize overall predictive performance (Vilalta and Drissi, 2002). The objective can be defined as:


$$\underset{\theta }{\min}\frac{1}{M}\overset{M}{\underset{i=1}{\sum }}L_{i}\left(\theta \right)$$

where θ represents meta-learner parameters, *M* is the number of tasks, and $L_{i}\left(\theta \right)$ is the task-specific loss.

**Figure 1:**
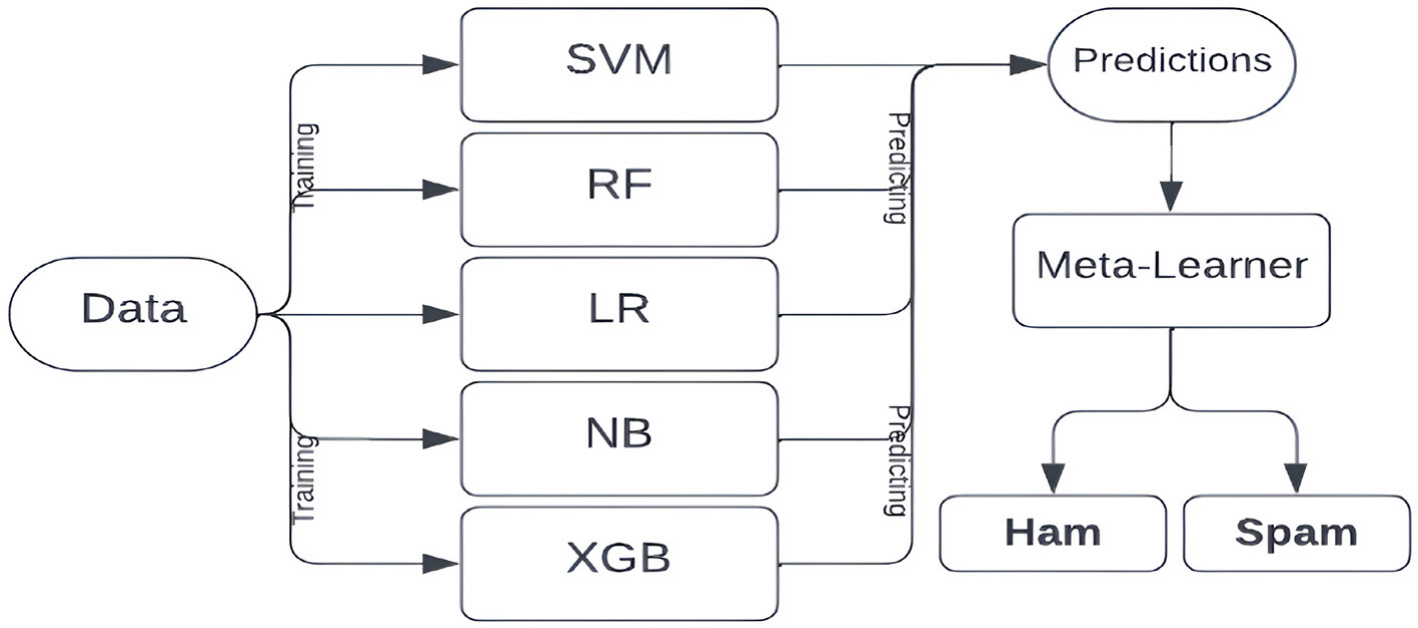
Flowchart of the proposed architecture for meta-learner.

For our spam-detection system, we design a logistic regression-based meta-learner trained on the predictions of five individual machine learning models, as illustrated in Figure 1. We intentionally exclude deep learning models since they are computationally more expensive and less interpretable, while traditional ML models perform better on relatively low-dimensional data like ours, where preprocessing and feature extraction reduce input complexity (Taye, 2023; Sharifani and Amini, 2023).

We include all five ML models rather than only top-performers to leverage complementary strengths and improve robustness. Random Forest and XGBoost, with their tree-based complexity, capture nuanced patterns, while simpler probabilistic models like Naive Bayes and Logistic Regression provide interpretable, stable predictions. Combining these diverse models mitigates overfitting and enhances overall performance (Fatima et al., 2023).

###### 2.2.1.1.1 XGBoost

eXtreme Gradient Boosting (XGBoost) is a supervised ensemble algorithm based on gradient boosting, designed for efficiency and scalability (Chen and Guestrin, 2016; Bacanin et al., 2022). It builds trees sequentially by fitting to the negative gradients (pseudo-residuals) of the loss function, with each new tree correcting errors from the previous iteration. A shrinkage factor helps control overfitting, and for our binary classification task, we use a logistic loss function. Final predictions are obtained by aggregating outputs from all trees in the ensemble.

###### 2.2.1.1.2 Random Forest

Random Forest is an ensemble algorithm that combines multiple decision trees to improve prediction accuracy (Kulkarni and Sinha, 2013). Each tree is trained on a bootstrapped dataset, and at each split, a random subset of features is considered. For our spam classification task, trees output binary predictions—spam or ham—and the final result is determined by majority vote. This bootstrap aggregation approach reduces variance and improves generalization. While less interpretable than a single decision tree, Random Forest mitigates overfitting effectively. Our model uses Gini impurity to select optimal splits by measuring misclassification likelihood (Yuan et al., 2021).

###### 2.2.1.1.3 Logistic regression

Logistic Regression is a widely used model for binary classification (Kleinbaum et al., 2010; Allison, 2012; Bacanin et al., 2022). It estimates the probability of belonging to the positive class using a sigmoid function and optimizes model parameters by minimizing cross-entropy loss. Gradient descent iteratively updates weights until convergence, and predictions are made by applying a 0.5 threshold on the output probability.

###### 2.2.1.1.4 Naive Bayes

Naive Bayes is a family of probabilistic models based on Bayes' Theorem, assuming conditional independence among features (Murphy, 2006). We use the Multinomial Naive Bayes variant, suitable for text classification with term frequency data. Each document is represented as a term-frequency vector, and the model computes class priors and term likelihoods. For new documents, posterior probabilities are calculated using Bayes' Theorem, and the class with the higher posterior is assigned.

###### 2.2.1.1.5 Support Vector Machine

Support Vector Machine (SVM) is a supervised learning algorithm effective for classification, particularly in high-dimensional or non-linear settings (Suthaharan and Suthaharan, 2016; Statnikov, 2011; Boswell, 2002). For binary classification, a linear SVM finds the optimal hyperplane that maximizes the margin between classes, using support vectors—the closest data points—to define the decision boundary. Maximizing this margin improves generalization and reduces overfitting, with the model trained via a constrained optimization problem reformulated using Lagrange multipliers.

##### 2.2.1.2 Vectorization approaches

###### 2.2.1.2.1 Bag of words

The Bag-of-Words (BoW) model is a feature extraction technique that represents documents as vectors of word frequencies, disregarding word order (Zhang et al., 2010). The dataset is tokenized to build a vocabulary of unique words, and each document is encoded as a vector whose length equals the vocabulary size, where each entry corresponds to the frequency of the associated word. This representation scales across the entire dataset to generate numerical features for modeling.

###### 2.2.1.2.2 Term frequency—inverse document frequency

TF-IDF is a vectorization method that measures the importance of a word within a document relative to the entire corpus (Ramos, 2003). Term Frequency (TF) quantifies how often a term appears in a document:


$$TF\left(t,d\right)=\frac{\text{Count of term }t\text{ in }d}{\text{Total terms in }d}$$

Inverse Document Frequency (IDF) measures how unique a term is across all documents:


$$IDF\left(t\right)=\log\left(\frac{N}{\text{Number of documents containing }t}\right)$$

The final score is computed as:


TF-IDF(t,d)=TF(t,d)·IDF(t)

Higher TF-IDF values indicate rarer, more informative terms, helping models focus on discriminative features.

#### 2.2.2 Deep learning system overview

##### 2.2.2.1 Models

###### 2.2.2.1.1 GRU and BiGRU

GRU, or Gated Recurrent Unit, is a type of recurrent neural network (RNN) designed to address the vanishing gradient problem in traditional RNNs. It uses two gates: an update gate, which controls how much of the previous hidden state to retain, and a reset gate, which determines how much past information to discard. These gates regulate the integration of the candidate state into the final hidden state, enabling GRUs to effectively capture long-range dependencies in sequential data (Dey and Salem, 2017; Gao and Glowacka, 2016). BiGRU, or Bidirectional GRU, extends this concept by processing sequences in both forward and backward directions. It combines the forward and backward hidden states through concatenation to form a comprehensive representation of contextual information, improving performance in tasks such as sentiment analysis and named entity recognition (Zhang et al., 2020; Cui et al., 2018).

###### 2.2.2.1.2 LSTM and BiLSTM

LSTM, or Long Short-Term Memory, is a recurrent neural network designed to address the limitations of traditional RNNs by incorporating three gates—input, forget, and output—along with a cell state for long-term memory and a hidden state for passing information between time steps (Staudemeyer and Morris, 2019; Gao and Glowacka, 2016). The gates regulate what information to store, discard, or output, enabling LSTMs to model long-term dependencies effectively, though the added parameters from the forget gate can increase the risk of overfitting. BiLSTM, or Bidirectional LSTM, extends this by processing sequences in both forward and backward directions. It concatenates forward and backward hidden states to form a comprehensive representation of contextual information, improving performance in natural language processing tasks by leveraging both past and future context.

###### 2.2.2.1.3 CNN

CNN, or Convolutional Neural Network, is a deep learning model designed for processing grid-like data such as images. It consists of convolutional layers, pooling layers, activation functions, and other components. Convolutional layers apply filters (kernels) to the input to generate feature maps, while pooling layers downsample spatial dimensions, reducing complexity and overfitting risk. Common pooling types include max pooling and average pooling. Activation functions like ReLU are applied element-wise to introduce non-linearity. Finally, flattening or global average pooling converts the extracted features into a one-dimensional vector for downstream processing (O'Shea and Nash, 2015; Zhang et al., 2020).

##### 2.2.2.2 Word embeddings

###### 2.2.2.2.1 Word embeddings vs. vectorization

Traditional vectorization methods like Bag-of-Words (BoW) and TF-IDF represent text as high-dimensional, sparse vectors, where each word corresponds to a dimension but semantic relationships are ignored (Singh and Shashi, 2019). For example, “king” and “queen” would be treated as unrelated. In contrast, word embeddings such as Word2Vec and GloVe produce dense, low-dimensional representations that capture semantic and contextual similarities by learning from surrounding words. These embeddings enable models to better understand language meaning, improving performance in tasks like text classification and sentiment analysis (Incitti et al., 2023).

###### 2.2.2.2.2 GloVe

GloVe, or Global Vectors for Word Representation, is an unsupervised algorithm for generating word embeddings (Pennington et al., 2014). It constructs a word co-occurrence matrix and optimizes an objective function that minimizes the difference between the dot product of embeddings and the logarithm of co-occurrence probabilities, using a weighting function to reduce the effect of rare pairs. Word vectors are trained with AdaGrad, producing embeddings that effectively capture semantic relationships.

###### 2.2.2.2.3 Word2Vec

Word2Vec is a self-supervised algorithm that learns word embeddings from surrounding context (Mikolov et al., 2013). After tokenizing text, a sliding window generates word-context pairs for training using either Continuous Bag of Words (CBoW), which predicts a word from its context, or Skip-gram, which predicts context from a word. The model is optimized via stochastic gradient descent, resulting in embeddings that represent semantic similarities between words.

##### 2.2.2.3 Principal component analysis

Principal Component Analysis (PCA) is a dimensionality reduction technique that projects high-dimensional data into a lower-dimensional space while preserving variance (Kuo and Sloan, 2005; Abdi and Williams, 2010). After standardizing the data, a covariance matrix is computed, and eigenvalue decomposition identifies principal components—eigenvectors corresponding to the largest eigenvalues. These components capture the most significant variance. In our work, PCA reduces the dimensionality of word embeddings, improving efficiency and mitigating overfitting while retaining essential semantic structure.

##### 2.2.2.4 Attention

Attention mechanisms enhance predictions by selectively focusing on relevant parts of an input sequence (Niu et al., 2021; Hernández and Amigó, 2021). In sequence-to-sequence tasks, an encoder processes the input, and the decoder generates the output using attention scores that measure the importance of each input element. These scores, computed via additive, dot-product, or multiplicative attention, are normalized with softmax to produce weights. A weighted sum of encoder states forms the context vector, guiding decoding. In our classification task, attention follows the same process with slight architectural adjustments for text classification.

### 2.3 Data collection and preprocessing

#### 2.3.1 Datasets

We use two publicly available benchmark datasets to build our email spam classification models, the Enron-Spam email dataset (Metsis et al., 2006) and TREC 2007 Public Corpus (Cormack, 2007). We train and evaluate our model on these two individual datasets, as well as a combined hybrid dataset. Additionally, to demonstrate the effectiveness of our meta-learner on recent emails, we use a newly released dataset for zero-shot evaluation (Miltchev et al., 2024):

- Enron-spam: the non-spam portion of the Enron-Spam dataset was collected during Enron's legal scandal. It consists of emails from the mailboxes of six specific employees: Louise Kitchen, Daren Farmer, Vincent Kaminski, Bill Williams, Sally Beck, and Michelle Lokay. The spam portion of the dataset was compiled from the following four sources: the SpamAssassin dataset, Project Honey Pot, spam emails collected from Bruce Guenter, and spam emails from Georgios Paliouras (one of the authors of Metsis et al., 2006). We combined these six smaller datasets into one larger dataset comprising 33,716 emails, of which 17,171 are spam.
- TREC 2007: TREC 2007 was presented in the Text Retrieval Conference (TREC) Spam Track of 2007 and has 75,419 total emails, of which 50,199 are spam. The conference focuses on information retrieval from large text datasets. The dataset was collected from a certain public server between April 8 and July 6, 2007, and includes all the emails the server received during that period with a few modifications. The server hosts numerous accounts that are no longer actively used but still receive significant amounts of spam. Among these, several “honeypot” accounts have been added, which are utilized to register for different services.
- Recent: the dataset was compiled by the University of Twente. It consists of 2,000 emails, half of which are ham emails and the rest are spam. It is composed of a mix of real-world emails and artificially generated ones.

The distribution of email types of the three datasets (and the hybrid dataset) is shown in Table 1, along with the 10 most frequent words for each dataset (after performing the first three steps of the data preprocessing techniques detailed in 2.3.2. Although the Enron-Spam and TREC 2007 datasets are dated, they remain benchmark datasets widely used in recent literature (Zavrak and Yilmaz, 2023; Adnan et al., 2024; Lee et al., 2023; Omotehinwa and Oyewola, 2023; Krishnamoorthy et al., 2024; Rabbi et al., 2023). Their continued relevance is also justified by their extensive size, providing a reliable source for model evaluation, and by the diversity of email sources they encompass, ensuring a less biased and more comprehensive representation of real-world spam and ham emails.

**Table 1:** Distribution of spam and non-spam emails for the two individual benchmark datasets, the hybrid (combined) dataset, and the real-world dataset, along with the 10 most frequent words in each category.

**Dataset**	**Spam**	**Non-spam**	**Total**	**Top 10 frequent words**
Enron	17,171	16,545	33,716	Ham: enron, ect, hou, company, say, please, would, com, subject, energy
				Spam: company, com, e, u, http, email, information, please, make, statement
TREC 2007	50,199	25,220	75,419	Ham: use, email, list, write, new, please, code, may, get, say
				Spam: contenttype, contenttransferencoding, pill, per, x, de, desjardins, price, quotedprintable, item
Hybrid	67,370	41,765	109,135	Ham: enron, ect, use, please, say, new, would, email, list, may
				Spam: contenttype, contenttransferencoding, pill, per, de, x, price, desjardins, quotedprintable, item
Recent	1,000	1,000	2,000	Ham: please, hi, dear, let, meet, next, find, attach, thank, week
				Spam: click, account, inform, please, review, avoid, subscript, renew, enjoy, payment

Other datasets mentioned in Section 2.1 which we did not use in our experiment include GenSpam (Medlock, 2005), SpamAssassin (Apache SpamAssassin, 2015), and LingSpam (Natural Language Processing Group, Department of Informatics - Athens University of Economics and Business, 2000), PU1 (Androutsopoulos et al., 2000), SpamBase (Hopkins et al., 1999), CSDMC2010 [(International Conference on Neural Information Processing (ICNIP), 2010)], and NewsGroup (Albishre et al., 2015).

#### 2.3.2 Data preprocessing

We performed data preprocessing on all the datasets to normalize the data before feeding it into our models and reduce unnecessary noise. Our preprocessing consisted of the following steps:

1. Cleaning: we removed all numbers, stop words, and special characters from the text. Stop words are considered insignificant because they don't add meaning to the text; they include words such as “a,” “from,” “this,” and “for.” Numbers and special characters are removed for similar reasons.
2. Lowercasing: to standardize the text and reduce vocabulary size, we convert all the characters to lowercase. This prevents the model from treating words with identical semantics as different (e.g. “meaning” vs. “Meaning”) because of different casing.
3. Lemmatization: this technique is used to reduce a word to its root form. Lemmatization is different from stemming because it reduces a word's suffix or prefix to its root, while lemmatization ensures the base word is linguistically valid. For instance, if we were to apply stemming, the word “changing” would be reduced to “chang,” whereas lemmatization would yield “change.”
4. Filtering: when training the models with GloVe embeddings, the dataset was filtered to exclude words not present in the GloVe database. Likewise, in the case of training with Word2Vec embeddings, the dataset underwent a similar preprocessing procedure in which words not found in the Word2Vec database were removed.

Table 2 displays an example of the text that would be preprocessed to improve model performance and reduce computation time.

**Table 2:** Example sentence transformation through preprocessing steps.

**Step**	**Sentence**
Original	The man (Srikar) finally walked home after a long day
Cleaned	Man Srikar finally walked home long day
Lowercased	Man srikar finally walked home long day
Lemmatized	Man srikar final walk home long day
Filtered^a^	Man final walk home long day
Final	Man final walk home long day

^a^GloVe embeddings were used in this example. The word “srikar” was removed because it is not in the GloVe vector database.

### 2.4 Experimental setup and meta-learner framework

This section outlines our complete experimental design for email spam classification. We build upon the model formulations (Section 2.2) and data preparation methods (Section 2.3) to compare various machine learning (ML) and deep learning (DL) models, and finally, we thoroughly describe the setup of our meta-learner.

#### 2.4.1 Overall methodology

**Figure 2:**
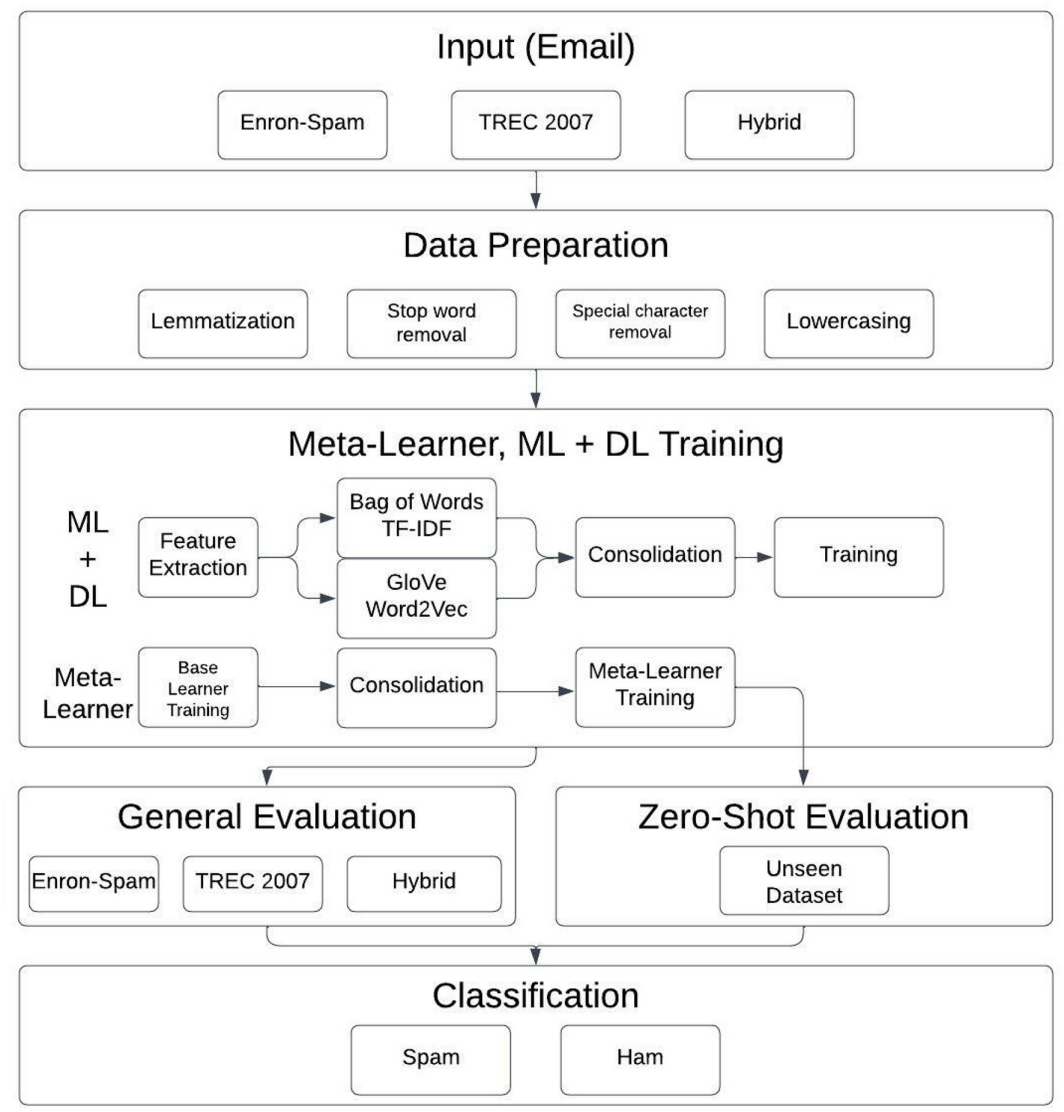
Flowchart of the proposed methodology for spam email classification.

- Preprocessing: all datasets were preprocessed via lemmatization, stop-word removal, and various other techniques.
- Pipeline structure: as shown in Figure 2, the cleaned data was input into:
  ° ML pipelines using TF-IDF and Bag-of-Words (BoW) vectorizers.
  ° DL pipelines using Word2Vec and GloVe embeddings, with and without attention mechanisms.
- Dataset variants:
  ° Evaluations were run on: the Enron-Spam dataset, the TREC 2007 dataset, and a hybrid dataset combining both.
  ° Using the hybrid dataset mitigated overfitting to specific email styles and enhanced generalization.

#### 2.4.2 Training and evaluation splits

- Machine learning models: 80% training/20% testing.
- Deep learning models: 60% training/20% validation/20% testing.
- Meta-learner:
  ° 60% of the data was used to train individual ML models.
  ° Predictions on the next 20% formed training data for the meta-learner.
  ° The final 20% was used to evaluate the meta-learner's performance.

#### 2.4.3 Deep learning architectures

**Figure 3:**
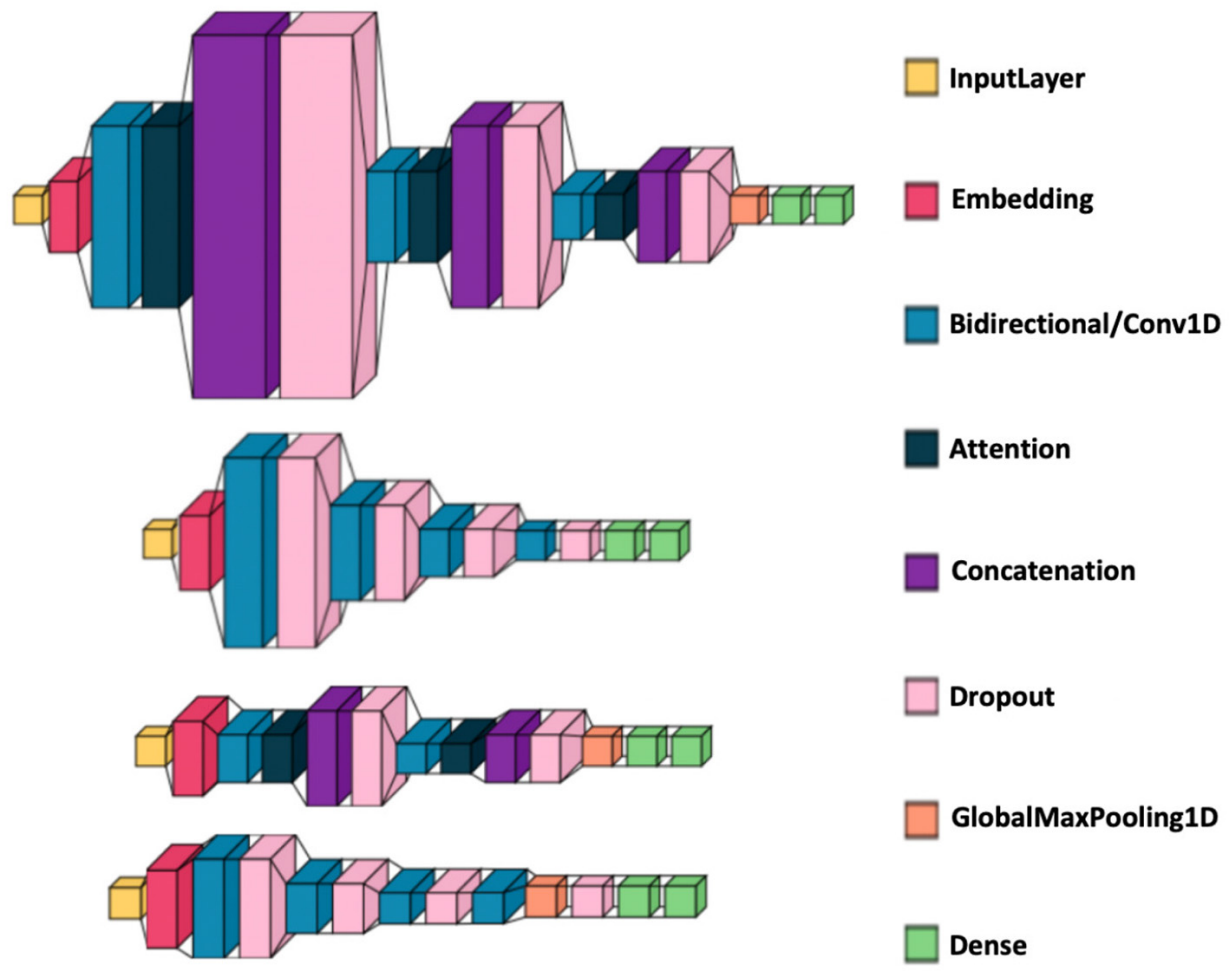
Deep learning model architecture visualizations. From top to bottom: BiLSTM with attention, BiLSTM without attention, CNN with attention, and CNN without attention. The medium blue color represents a Bidirectional layer for the BiLSTM models and a Conv1D layer for the CNN models.

- Without attention: each model had four layers with 128, 64, 32, and 16 units (including dropout), followed by two dense layers (16 units and 1 unit).
- With attention: each model consisted of three layers (128, 64, 32 units) followed by attention, concatenation, dropout, GlobalAveragePooling, and two dense layers (16 and 1 units).
- CNN specifics: incorporated a GlobalAveragePooling layer between the convolutional core and the dense output layers.
- Figure 3 illustrate the visualizations of the deep learning architectures.

#### 2.4.4 Word embedding approaches

- GloVe: used 100-dimensional pretrained vectors; all words not in the GloVe vocabulary were removed.
- Word2Vec: publicly available 300-dimensional vectors were reduced to 100 dimensions using Principal Component Analysis (PCA) for fair comparison with GloVe (see Section 2.2.2.3).

#### 2.4.5 Meta-learner architecture

##### 2.4.5.1 Base model preparation

- Five ML models (XGBoost, Random Forest, SVM, Naive Bayes, Logistic Regression) were trained on 60% of the hybrid dataset.
- Predictions on the subsequent 20% validation split served as meta-features for the stacking model.
- Each ML model was benchmarked under both TF-IDF and BoW; only the higher-performing vectorizer per model was retained for meta-training.

##### 2.4.5.2 Meta-model training and inference

- A logistic regression model was employed as the meta-learner.
- It was trained on the concatenated prediction outputs of the five base ML models.
- The final evaluation was performed on the held-out 20% test split.

#### 2.4.6 Zero-shot evaluation

- Base ML models were retrained using 70% of the hybrid dataset.
- Predictions on the remaining 30% were used to generate meta-training features.
- Following prior work in zero-shot spam detection (Shirvani and Ghasemshirazi, 2025; Rojas-Galeano, 2024), we evaluate our meta-learner directly on an unseen email corpus of 2,000 emails introduced by Miltchev et al. (2024) without any additional fine-tuning to assess out-of-domain generalization capabilities.
- We follow the core meta-learning paradigm of Liu et al. (2021) in their TGMZ model. Like TGMZ, we train our meta-learner on one data distribution and then apply it directly to a fully unseen email corpus without any fine-tuning. This episodic evaluation, training on one “task” and testing on another unseen one, embodies zero-shot generalization. This setup is similarly explored in Verma et al. (2020).

## 3 Results

Through extensive experimentation and analysis, we found that the meta-learner built on machine learning (ML) models' predictions consistently outperformed individual deep learning models. This result led us to conclude that the ML-based meta-learner was effectively capturing important patterns in the data that deep learning models were unable to capture as efficiently. By leveraging the strengths of machine learning, we achieved significant improvements in spam classification accuracy without introducing the additional complexity associated with deep learning-based meta-learning techniques.

We now present the detailed results of our machine-learning and deep-learning pipelines on the four datasets. The classification metrics used to evaluate the models are accuracy, precision, recall, F-score, and AUC. These metrics are formulated based on the concepts of True Positives (TP)—correctly predicting the positive class, True Negatives (TN)—correctly predicting the negative class; False Positives (FP)—incorrectly predicting the positive class; and False Negatives (FN)—incorrectly predicting the negative class. For formal definitions and comparisons of these metrics, please refer to Obi (2023).

All metrics presented for the machine learning models use five-fold cross-validation. The metrics accuracy, precision, recall, and F-score in the tables and visuals will be abbreviated to their first letter. The meta-learner metrics will be italicized and placed as the last row for each benchmark dataset's machine learning pipelines table.

### 3.1 Enron-spam dataset results

We will start by presenting the machine learning and deep learning pipeline results on the Enron-Spam dataset. Table 3A shows how Random Forest with TF-IDF obtained the highest average accuracy of 0.9796. Furthermore, of all 10 pipelines, Random Forest (including both vectorization approaches) performed the best for four out of the five metrics.

**Table 3A:** Machine learning pipelines performance metrics on Enron-Spam dataset.

**Model**	**Vectorization**	**A**	**P**	**R**	**F**	**AUC**
XGBoost	BOW	0.9656	0.9439	0.9911	0.9668	0.9653
	TF-IDF	0.9651	0.9426	0.9915	0.9663	0.9648
Random Forest	BOW	0.9780	**0.9724**	0.9845	0.9784	0.9780
	TF-IDF	**0.9796**	0.9696	0.9907	**0.9800**	**0.9795**
Logistic Regression	BOW	0.9792	0.9680	0.9917	0.9797	0.9791
	TF-IDF	0.9724	0.9521	0.9957	0.9733	0.9721
Naive Bayes	BOW	0.9734	0.9642	0.9843	0.9740	0.9733
	TF-IDF	0.9780	0.9711	0.9860	0.9784	0.9779
Support Vector Machine	BOW	0.9505	0.9179	0.9913	0.9530	0.9501
	TF-IDF	0.9770	0.9595	**0.9967**	0.9777	0.9768
Meta-learner	–	*0.9898*	*0.9898*	*0.9898*	*0.9898*	*0.9991*

Bold values indicate the top score for each metric across traditional ML models. Italic values indicate the top score for each metric across all models (traditional ML and meta-learner).

**Figure 4:**
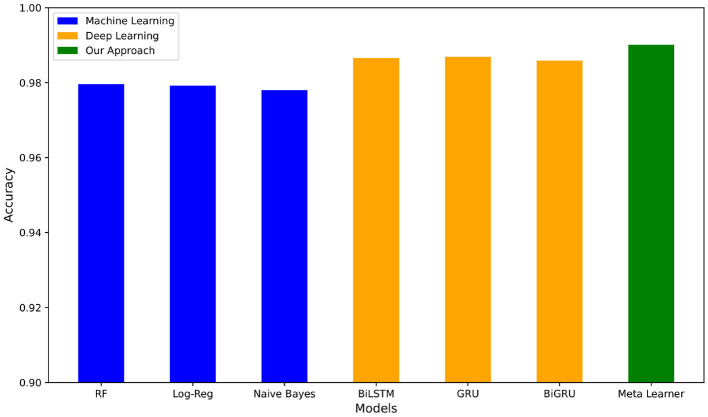
Accuracy of best ML and DL pipelines vs. our meta-learner on Enron-Spam dataset.

For the deep learning models as shown in Table 3B, the two best values per metric are bolded. We see that BiLSTM-Attention-GloVe and GRU-Attention-GloVe performed the best for the first four metrics (accuracy, precision, recall, F-score), while BiGRU-Attention-GloVe and CNN-No Attention-GloVe had the highest AUC scores. The meta-learner outperformed all ML and DL model performances (Tables 3A,B) with an accuracy of 0.9898. Figure 4 presents the accuracies of the top ML and DL models and demonstrates that the meta-learner achieves superior performance. Table 3C displays the confusion matrix for the meta-learner. The accuracy for the Ham (0) class is $\frac{3,291}{3,291+48}\approx 0.9856$ and the accuracy for the Spam (1) class is $\frac{3,297}{3,297+20}\approx 0.9940.$

**Table 3B:** Deep learning pipelines model performance metrics on Enron-Spam dataset.

**Model**	**Attention**	**Word embedding**	**A**	**P**	**R**	**F**	**AUC**
LSTM	Without	GloVe	0.9847	0.9847	0.9847	0.9847	0.9984
	With	GloVe	0.9857	0.9858	0.9857	0.9857	0.9975
	Without	Word2Vec	0.9833	0.9833	0.9833	0.9833	0.9970
	With	Word2Vec	0.9851	0.9851	0.9851	0.9851	0.9977
BiLSTM	Without	GloVe	0.9856	0.9856	0.9856	0.9856	0.9975
	With	GloVe	**0.9866**	**0.9867**	**0.9866**	**0.9866**	0.9982
	Without	Word2Vec	0.9847	0.9847	0.9847	0.9847	0.9965
	With	Word2Vec	0.9832	0.9832	0.9832	0.9832	0.9979
GRU	Without	GloVe	0.9827	0.9828	0.9827	0.9827	0.9975
	With	GloVe	**0.9869**	**0.9869**	**0.9869**	**0.9869**	0.9976
	Without	Word2Vec	0.9781	0.9781	0.9781	0.9781	0.9966
	With	Word2Vec	0.9844	0.9844	0.9844	0.9844	0.9985
BiGRU	Without	GloVe	0.9859	0.9860	0.9859	0.9859	0.9983
	With	GloVe	0.9854	0.9857	0.9854	0.9854	**0.9992**
	Without	Word2Vec	0.9853	0.9853	0.9853	0.9853	0.9980
	With	Word2Vec	0.9851	0.9851	0.9851	0.9851	0.9977
CNN	Without	GloVe	0.9848	0.9850	0.9848	0.9848	**0.9987**
	With	GloVe	0.9790	0.9791	0.9790	0.9790	0.9978
	Without	Word2Vec	0.9814	0.9814	0.9814	0.9814	0.9987
	With	Word2Vec	0.9823	0.9825	0.9823	0.9823	0.9983

Bold values indicate the top two scores for each metric.

**Table 3C:** Confusion matrix for the meta-learner on Enron-Spam dataset.

	**Predicted**	
**Actual**	**0 (Ham)**	**1 (Spam)**
0 (Ham)	3,291	48
1 (Spam)	20	3,297

### 3.2 TREC 2007 dataset results

**Figure 5:**
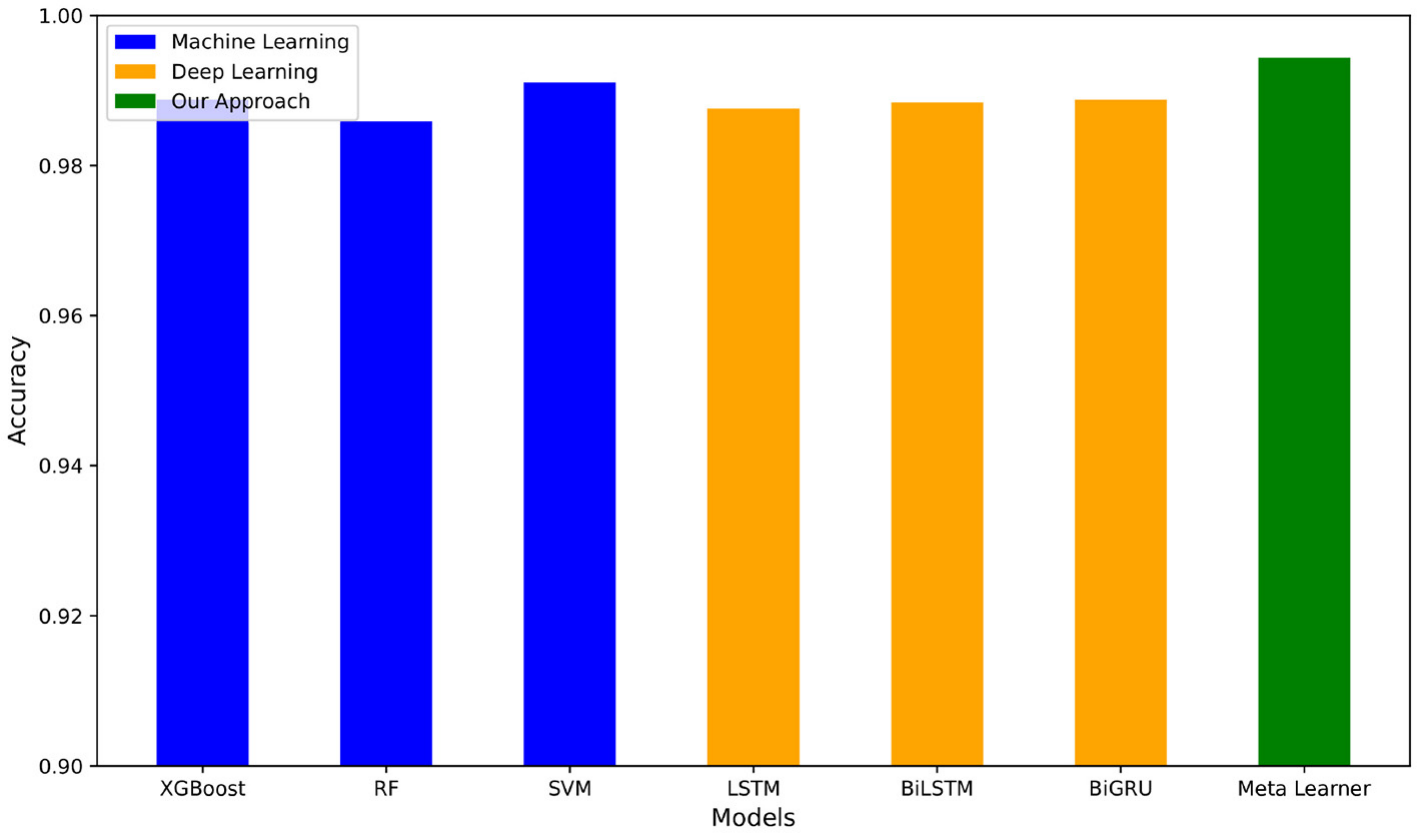
Accuracy of best ML and DL pipelines vs. our meta-learner on TREC 2007 dataset.

For the TREC 2007 dataset, Table 4a shows that Support Vector Machine with TF-IDF performed the best with an average accuracy of 0.9911, while Naive Bayes with Bag of Words had the lowest average accuracy of 0.9378. For the deep learning pipelines shown in Table 4b, BiLSTM-Attention-GloVe and BiGRU-Attention-GloVe had the best performances with accuracies of 0.9884 and 0.9888, respectively. The highest accuracy of all models was our meta-learner with 0.9945. Figure 5 compares our meta-learner with the accuracies of the top ML and DL models. Table 4c displays the confusion matrix for the meta-learner. The accuracy for the Ham (0) class is $\frac{5,029}{5,029+50}\approx 0.9902$ and the accuracy for the Spam (1) class is $\frac{9,598}{9,598+31}\approx 0.9968$.

Table 4Performance metrics on TREC 2007 dataset.

**Table:** 

**(a) Machine learning pipelines performance metrics on TREC 2007 dataset**						
**Model**	**Vectorization**	**A**	**P**	**R**	**F**	**AUC**
XGBoost	BOW	0.9888	0.9900	0.9930	0.9915	0.9869
	TF-IDF	0.9883	0.9897	0.9926	0.9911	0.9863
Random Forest	BOW	0.9859	0.9936	0.9849	0.9892	0.9864
	TF-IDF	0.9856	0.9932	0.9849	0.9890	0.9860
Logistic Regression	BOW	0.9845	0.9873	0.9892	0.9882	0.9824
	TF-IDF	0.9852	0.9847	0.9929	0.9888	0.9816
Naive Bayes	BOW	0.9378	0.9959	0.9091	0.9504	0.9509
	TF-IDF	0.9671	0.9885	0.9612	0.9746	0.9698
Support Vector Machine	BOW	0.9753	0.9760	0.9867	0.9813	0.9701
	TF-IDF	**0.9911**	**0.9906**	**0.9960**	**0.9933**	**0.9889**
Meta-learner	-	*0.9945*	*0.9945*	*0.9935*	*0.9939*	*0.9990*

**Table:** 

**(b) Deep learning model performance metrics on TREC 2007 dataset**							
**Model**	**Attention**	**Word embedding**	**A**	**P**	**R**	**F**	**AUC**
LSTM	Without	GloVe	0.9677	0.9688	0.9677	0.9679	0.9952
	With	GloVe	0.9876	0.9876	0.9876	0.9876	0.9970
	Without	Word2Vec	0.9789	0.9790	0.9789	0.9789	0.9955
	With	Word2Vec	0.9865	0.9865	0.9865	0.9865	0.9975
BiLSTM	Without	GloVe	0.9851	0.9851	0.9851	0.9851	0.9973
	With	GloVe	**0.9884**	**0.9884**	**0.9884**	**0.9884**	**0.9981**
	Without	Word2Vec	0.9814	0.9816	0.9814	0.9815	0.9960
	With	Word2Vec	0.9854	0.9854	0.9854	0.9853	0.9978
GRU	Without	GloVe	0.9727	0.9733	0.9727	0.9728	0.9963
	With	GloVe	0.9867	0.9868	0.9867	0.9868	0.9972
	Without	Word2Vec	0.9852	0.9852	0.9852	0.9852	0.9971
	With	Word2Vec	0.9852	0.9852	0.9852	0.9852	0.9971
BiGRU	Without	GloVe	0.9855	0.9855	0.9855	0.9855	0.9974
	With	GloVe	**0.9888**	**0.9889**	**0.9888**	**0.9889**	**0.9976**
	Without	Word2Vec	0.9844	0.9844	0.9844	0.9843	0.9979
	With	Word2Vec	0.9851	0.9852	0.9851	0.9851	0.9973
CNN	Without	GloVe	0.9838	0.9838	0.9838	0.9838	0.9967
	With	GloVe	0.9842	0.9842	0.9842	0.9842	0.9974
	Without	Word2Vec	0.9837	0.9839	0.9837	0.9838	0.9977
	With	Word2Vec	0.9852	0.9852	0.9852	0.9852	0.9972

**Table:** 

**(c) Confusion matrix for the meta-learner on TREC 2007 Dataset**		
	**Predicted**	
**Actual**	**0 (Ham)**	**1 (Spam)**
0 (Ham)	5,029	50
1 (Spam)	31	9,598

(a) Bold values indicate the top score for each metric across traditional ML models. (b) Bold values indicate the top two scores for each metric. (a) Italic values indicate the top score for each metric across all models (traditional ML and meta-learner).

### 3.3 Hybrid dataset results

**Figure 6:**
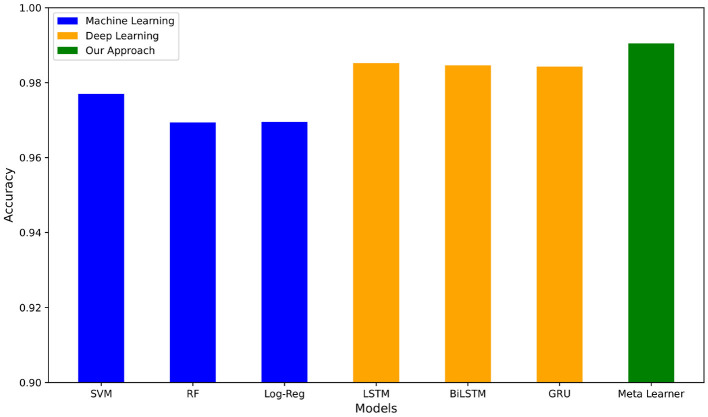
Accuracy of best ML and DL pipelines vs. our meta-learner on hybrid dataset.

We now present results on the hybrid dataset, consisting of the combination of Enron-Spam and TREC 2007. For the machine learning pipelines in Table 5a, Support Vector Machine with TF-IDF has the highest average accuracy of 0.9773 and the highest recall, F1-score, and AUC score. Naive Bayes with Bag of Words has the highest precision. Of the deep learning pipelines in Table 5b, LSTM-Attention-GloVe and BiLSTM-Attention-GloVe are the best performing with accuracies and F-scores of 0.9852 and 0.9846, respectively. Our meta-learner outperforms all pipelines with an accuracy of 0.9904, an F-score of 0.9899, and an AUC score of 0.9991. Figure 6 illustrates the accuracies of the best-performing ML and DL models, highlighting that the meta-learner surpasses both. Table 5c displays the confusion matrix for the meta-learner. The accuracy for the Ham (0) class is $\frac{8,284}{8,284+124}\approx 0.9853$ and the accuracy for the Spam (1) class is $\frac{12,873}{12,873+82}\approx 0.9937.$

Table 5Performance metrics on hybrid dataset.

**Table:** 

**(a) Machine learning pipelines performance metrics on hybrid dataset**						
**Model**	**Vectorization**	**A**	**P**	**R**	**F**	**AUC**
XGBoost	BOW	0.9610	0.9513	0.9878	0.9690	0.9535
	TF-IDF	0.9612	0.9521	0.9872	0.9691	0.9539
Random Forest	BOW	0.9685	0.9791	0.9691	0.9741	0.9683
	TF-IDF	0.9694	0.9752	0.9750	0.9750	0.9678
Logistic Regression	BOW	0.9695	0.9653	0.9858	0.9754	0.9650
	TF-IDF	0.9689	0.9613	0.9893	0.9750	0.9631
Naive Bayes	BOW	0.9436	**0.9870**	0.9197	0.9518	0.9504
	TF-IDF	0.9612	0.9755	0.9604	0.9678	0.9614
Support Vector Machine	BOW	0.9467	0.9348	0.9835	0.9580	0.9421
	TF-IDF	**0.9773**	0.9711	**0.9927**	**0.9817**	**0.9729**
Meta-learner	-	*0.9904*	*0.9904*	*0.9895*	*0.9899*	*0.9991*

**Table:** 

**(b) Deep learning model performance metrics on hybrid dataset**							
**Model**	**Attention**	**Word embedding**	**A**	**P**	**R**	**F**	**AUC**
LSTM	Without	GloVe	0.9826	0.9828	0.9826	0.9826	0.9980
	With	GloVe	**0.9852**	**0.9852**	**0.9852**	**0.9852**	**0.9985**
	Without	Word2Vec	0.9795	0.9795	0.9795	0.9795	0.9970
	With	Word2Vec	0.9816	0.9816	0.9816	0.9816	0.9975
BiLSTM	Without	GloVe	0.9810	0.9810	0.9810	0.9810	0.9972
	With	GloVe	**0.9846**	**0.9845**	**0.9846**	**0.9846**	**0.9978**
	Without	Word2Vec	0.9779	0.9780	0.9779	0.9780	0.9957
	With	Word2Vec	0.9811	0.9812	0.9811	0.9811	0.9977
GRU	Without	GloVe	0.9742	0.9742	0.9742	0.9741	0.9961
	With	GloVe	0.9843	0.9843	0.9843	0.9843	0.9978
	Without	Word2Vec	0.9689	0.9689	0.9689	0.9688	0.9938
	With	Word2Vec	0.9842	0.9842	0.9842	0.9842	0.9973
BiGRU	Without	GloVe	0.9607	0.9615	0.9607	0.9605	0.9924
	With	GloVe	0.9756	0.9757	0.9756	0.9756	0.9968
	Without	Word2Vec	0.9693	0.9693	0.9693	0.9693	0.9936
	With	Word2Vec	0.9746	0.9750	0.9746	0.9745	0.9965
CNN	Without	GloVe	0.9818	0.9818	0.9818	0.9818	0.9972
	With	GloVe	0.9800	0.9800	0.9800	0.9800	0.9965
	Without	Word2Vec	0.9798	0.9799	0.9798	0.9798	0.9970
	With	Word2Vec	0.9785	0.9785	0.9785	0.9784	0.9963

**Table:** 

**(c) Confusion matrix for the meta-learner on hybrid dataset**		
	**Predicted**	
**Actual**	**0 (Ham)**	**1 (Spam)**
0 (Ham)	8,284	124
1 (Spam)	82	12,873

(a) Bold values indicate the top score for each metric across traditional ML models. (b) Bold values indicate the top two scores for each metric. (a) Italic values indicate the top score for each metric across all models (traditional ML and meta-learner).

#### 3.3.1 Meta-learner interpretability on hybrid dataset

The hybrid dataset combines the strengths of two widely used datasets, Enron-Spam and the TREC 2007, making it a suitable testbed for both accuracy and interpretability claims. Our meta-learner treats each base model's posterior as an input feature; specifically, for the hybrid dataset the five “features” are *svm_tfidf* , *xgb_tfidf*, *rf_tfidf*, *nb_tfidf*, *lr_count*, which was determined based on empirical evaluations of the individual models on the dataset. This architecture supports a clear, named, low-dimensional feature space.

Using permutation importance (Altmann et al., 2010) with ROC-AUC as the score on the held-out test split, we observe the largest mean performance drop when permuting *svm_tfidf* (≈0.0128 AUC), with smaller but non-trivial drops for *rf_tfidf* (≈0.0031) and *xgb_tfidf* (≈0.0028). This ranking indicates that the stacker's discriminative power is primarily mediated by the SVM-TF-IDF base learner.

A complementary SHAP analysis (Lundberg and Lee, 2017) of the meta-learner yields consistent rankings. The top three features by global importance (mean |SHAP|) are: *svm_tfidf* = 2.237, *xgb_tfidf* = 1.536, and *rf_tfidf* = 1.505. These three account for ≈89.6% of total mean |SHAP|, indicating that the meta-learner's decisions are chiefly mediated by the SVM–TF–IDF posterior with complementary signal from the TF–IDF tree models.

We also audit the dominant base learner (*svm_tfidf* ) with a LIME-Text (Ribeiro et al., 2016) pass on a stratified sample of 600 test emails. The *Phishing*-pushing side is dominated by actionable and credential/brand cues (e.g., *click, secure, info*, along with organization or domain markers such as *inc* and *com*). Conversely, tokens characteristic of legitimate organizational/news traffic and routine workflows (e.g., *bbc, attached*/*attach, enron, unsubscribe*/*subscribe*) systematically pull toward *Ham*.

### 3.4 Comparison to state of the art

We compare the performance of our meta-learners to other state-of-the-art models. In the Dataset column of Table 6, “EN” refers to the Enron-Spam dataset, “TR” to TREC 2007, and “SA” to SpamAssassin. The column “Instances” refers to the total number of email instances in the dataset. The last row, whose dataset is labeled as “Recent,” refers to the meta-learner's performance on the unseen recent dataset (Miltchev et al., 2024). All selected state-of-the-art models have been published within the last four years and experimentation involved at least one of the datasets Enron-Spam or TREC.

**Table 6:** Comparison of the proposed approach vs. state-of-the-art models.

**References**	**Year**	**Dataset**	**Instances**	**Method**	**A**	**P**	**R**	**F**	**AUC**
Zavrak and Yilmaz (2023)	2023	EN	33,654	Hybrid NN	0.958	0.981	0.937	0.958	0.989
		TR	75,288		0.992	0.989	0.999	0.994	0.999
Guo et al. (2023)	2022	EN	33,716	BERT + Log-Reg	–	0.9786	0.9783	0.9784	0.9971
Tida and Hsu (2022)	2022	EN	32,638	BERT	0.97	0.96	0.98	0.9720	–
Wang et al. (2021)	2021	EN	33,702	SVM	0.939	–	–	–	–
Adnan et al. (2024)	2023	EN+SA	13,629	ML meta-learner	0.988	0.988	0.989	0.989	–
Ezpeleta et al. (2020)	2020	TR	75,419	Log-Reg	0.9918	–	–	–	–
Lee et al. (2023)	2023	EN	33,716	Naive Bayes	0.9708	0.9703	0.9721	0.9712	–
		TR	75,419		0.9597	0.9557	0.9855	0.9701	–
Chu et al. (2020)	2020	EN	7,800	C4.5 Algorithm	0.9859	–	0.9779	–	–
		TR	10,000		0.9892	–	0.9808	–	–
Omotehinwa and Oyewola (2023)	2023	EN	32,860	XGBoost	0.9809	0.9748	0.9884	0.9816	0.9978
Krishnamoorthy et al. (2024)	2024	EN	33,727	DNN-BiLSTM	0.9869	0.9883	0.9856	0.9869	–
Ghogare et al. (2023)	2023	EN	46,932	Random Forest	0.9869	0.9876	0.9795	0.9835	–
Chirra et al. (2020)	2020	EN	6,000	CNN	0.985	–	–	–	–
Rabbi et al. (2023)	2023	TR	75,419	Random Forest	0.9838	0.9840	0.9838	0.9838	0.9820
**Our approach**	**2024**	**EN**	**33,716**	**Meta-learner**	**0.9898**	**0.9898**	**0.98**	**0.9898**	**0.9991**
		**TR**	**75,419**		**0.9945**	**0.9945**	**0.9935**	**0.9939**	**0.9990**
		**EN + TR**	**109,135**		**0.9904**	**0.9904**	**0.9895**	**0.9899**	**0.9991**
		**Recent**	**2,000**		**0.6340**	**0.6853**	**0.6340**	**0.6068**	**0.7605**

Bold values indicate the top score for each metric, by dataset.

Table 6 shows how our Enron-Spam and TREC 2007 meta-learners outperform all other SOTA approaches with both accuracies and F-scores of 0.9901 and 0.9944, respectively. The model tested on Enron-Spam with the closest performance to our model is proposed by Adnan et al. (2024) with an accuracy of 0.988. Furthermore, for models tested on TREC 2007, the closest accuracy to our model is 0.992 by Zavrak and Yilmaz (2023). The strong performance of our meta-learner models demonstrates their robustness on diverse datasets.

Finally, we find that our meta-learner outperforms the only other meta-learning-based spam classifier we identified, proposed by Adnan et al. (2024), achieving an accuracy of 0.9905 compared to their 0.988. Notably, our model was trained on a hybrid dataset over eight times larger, enabling better generalization and robustness across diverse email distributions. It also maintains lower computational complexity and explicitly addresses data bias, helping reduce downstream algorithmic bias. These advantages make our meta-learning pipeline not only more accurate, but also more practical and scalable for real-world spam classification deployments.

The meta-learner trained on the hybrid dataset was then zero-shot evaluated on a recent real-world dataset, following practices of Verma et al. (2020) and Liu et al. (2021), exhibiting an accuracy of 0.6340 and an F-score of 0.6068, with a spam true positive rate (TPR) of 0.8970. Achieving such high spam TPR on a recent dataset without any fine-tuning indicates that our meta-learner can effectively identify phishing and spam patterns even in different email environments. Moreover, the high zero-shot performance demonstrates that our hybrid dataset effectively captures diverse spam patterns, enabling the model to transfer its knowledge to unseen data. A confusion matrix can be seen in Table 7. The accuracy for the Ham (0) class is $\frac{371}{371+629}=0.3710$ and the accuracy for the Spam (1) class is $\frac{897}{897+103}=0.8970.$

**Table 7:** Final meta-learner confusion matrix.

	**Predicted**	
**Actual**	**0 (Ham)**	**1 (Spam)**
0 (Ham)	371	629
1 (Spam)	103	897

## 4 Discussion

### 4.1 Analysis of trends in results

We now evaluate some common trends apparent in the results of the various machine learning and deep learning pipelines and the comparison to state-of-the-art models:

1. Word embeddings: for the hybrid dataset combining Enron-Spam and TREC 2007, the deep learning pipeline with GloVe embeddings outperformed the pipeline with Word2Vec embeddings, in terms of accuracy, for nine out of 10 instances. Similar trends occurred with the individual datasets as GloVe performed better for nine out of 10 cases on Enron-Spam and seven out of 10 on TREC 2007. We suspect this is the case because when filtering each dataset to only include words from either of these two word embedding vocabularies, the size of the filtered dataset was larger for Word2Vec than for GloVe. This suggests that Word2Vec has more vocabulary relevant to our specific use case, spam email classification. Furthermore, using Principal Component Analysis on the Word2Vec embeddings to transform the representations from 300 dimensions to 100 dimensions may have slightly diluted the quality of these representations, thus limiting the performance of the models on the datasets.
2. Attention: deep learning architectures with attention mechanisms generally outperform identical architectures without them because attention focuses on the most relevant parts of the input, reducing information dilution.
3. Vectorization: TF-IDF vectorization pipelines generally outperformed Bag of Word pipelines for the machine learning models. This was the case for four out of five instances on the hybrid dataset and three out of five instances for the individual datasets, Enron-Spam and TREC 2007.
4. Models: we first note that BiLSTM consistently appears among the two highest-performing models in terms of accuracy for each set of 20 deep learning pipelines across the three main datasets. The Support Vector Machine performed the best of the machine learning odels.
5. Meta-learner: our proposed approach with a meta-learner combining the predictions of the individual machine learning models outperforms all other pipelines evaluated and state-of-the-art models. Compared to many of the deep learning-based methods used in SOTA, our approach offers a significant advantage in terms of complexity. Deep learning models, especially those with attention mechanisms or extensive architectures like transformers, can be computationally expensive to train and deploy. They require substantial computational resources, including powerful GPUs and large amounts of memory. In contrast, our meta-learner approach aggregates simpler models and tends to be less computationally intensive, making it more accessible and cost-effective to implement. This improves our model's scalability and speed, thus making it more effective in real use cases.
6. Bias: in our research, we evaluated various models on two benchmark datasets and also combined them to form a hybrid dataset. Furthermore, we tested our developed meta-learner on a medium-size unseen and recent dataset. This differs from most SOTA approaches, which only evaluate performance on a single benchmark dataset. Integrating multiple datasets mitigates the bias inherent in single-dataset evaluations, leading to a more robust and generalizable model. This ensures that our meta-learner model can perform well across different data distributions, enhancing its applicability to a wider range of real-world scenarios; we thus address both data bias and algorithmic bias by providing a more balanced and comprehensive evaluation.
7. Real-world use cases: as discussed in 3.4 our meta-learner yields an accuracy of 0.6320 when evaluated in a zero-shot setting. While this performance is evidently numerically lower than on the benchmark datasets where training data was available; we argue that it provides crucial insights into the meta-learner's robustness and practical utility in dynamic, real-world email environments.
  (a) *Operational value*: the confusion matrix in Table 6 reveals class-specific accuracies of 0.3710 (Ham) and 0.8970 (Spam). This asymmetric performance is often desirable in security-focused spam detection algorithms. In particular, the model demonstrates high recall on the positive class (spam)—a crucial metric, since false negatives are more costly than false positives. A missed spam email could expose users to phishing or malware attacks, whereas a false positive merely results in a benign email being misclassified.
  (b) *Computational efficiency and constraints:* unlike transformer-based models or deep hybrid attention mechanisms, our meta-learner is built on lightweight machine learning classifiers. It is trainable in CPU hardware in minutes, highly interpretable, and easily deployable. Commercial email providers like Gmail or Outlook often tout high spam detection accuracy and minimal false negative rates, but these systems are trained using massive, ever-growing streams of user data. With access to millions of emails per day and real-time behavioral feedback (e.g., user flagging, engagement patterns), they leverage continual learning and large-scale infrastructure to maintain performance against evolving spam techniques. In contrast, our meta-learner was trained once, using a finite and well-curated dataset totaling approximately 100,000 emails, and yet it achieved strong results—including a zero-shot accuracy of 0.6340 on a modern unseen dataset—without any retraining or tuning. This highlights the practicality and generalizability of our approach.
8. Zero-shot evaluation on a recent dataset: We evaluate our hybrid meta-learner zero-shot on a recent public dataset of 2,000 emails labeled as Safe or Phishing. We select this dataset because it is recent, openly accessible, and explicitly released for validating email classifiers without special access requirements.
  (a) On the recent dataset used in our study, there are 1,000 Safe and 1,000 Phishing emails. Messages are short (mean 86.7 characters, 14.1 tokens). Phishing emails are shorter on average (82.9 characters, 12.9 tokens) than Safe emails (90.6, 15.3). All messages are ASCII and no URLs or HTML tags appear in the dataset. Also, the combined vocabulary is small (174 tokens), with substantial overlap across classes.
  (b) These properties differ from Enron-Spam and TREC 2007. Enron-Spam consists of natural emails and “fresh” spam distributed across six user mailboxes and is widely used for filter evaluation. TREC 2007 is a large, chronologically ordered email stream (75,419 messages). Both datasets are larger, more heterogeneous, and include richer email structure than the recent dataset.
  (c) These differences help explain our zero-shot pattern on the recent dataset (accuracy 0.6340, F-score 0.6068, spam TPR 0.8970). First, the task framing differs as the recent dataset targets phishing specifically, whereas the benchmark datasets evaluate spam broadly. Second, short, template-like texts with no URLs/HTML reduce discriminative cues that modern systems often rely on. Third, the balanced class prior and short-message style shift the optimal decision threshold relative to our benchmark-trained operating point. Together, these factors lead to high recall on the positive class and lower accuracy on the ham class.
  (d) A few improvements for future iterations include: 1. Re-tune the operating threshold on precision–recall curves for the recent dataset and report the chosen point, 2. Add a small set of non-lexical features available at inference (e.g., message length statistics, simple style markers, and, when available, header or URL indicators), 3. Perform light domain adaptation using unlabeled recent emails (importance reweighting of benchmark dataset training instances before refitting the base learners and the meta-learner), 4. Label a small, diverse subset of recent emails (prioritizing distinct templates) and fine-tune, and 5. Adopt a periodic refresh schedule (continual learning) with dated external datasets to track evolving email patterns.

### 4.2 Future steps

In this study, we compare traditional machine learning and deep learning models. Given the recent popularity of artificial intelligence, we plan to evaluate the performances of transformer-based models in the future. In particular, we plan to conduct a similar study on the state-of-the-art NLP models, including BERT and XLNet. Furthermore, we will compare the performances of different Large Language Models (LLMs), including GPT, Gemini, and Llama.

In this study, we evaluated different machine learning vectorization approaches and deep learning word embedding approaches and architectures. In future studies, we plan to experiment with different data splits and hyperparameters instead to optimize models without significantly increasing complexity. Hyperparameter tuning will be conducted using methods such as grid search and random search to identify the optimal parameters for each model, balancing performance with computational efficiency.

Finally, we plan to explore different feature extraction techniques. Many recent studies have evaluated methods in which certain features from the dataset were used to train models, such as the number of special characters per email, email length, presence of certain keywords, and topic modeling. These approaches may provide greater generalizability, which is something worth researching.

## Data Availability

The raw data supporting the conclusions of this article will be made available by the authors, without undue reservation.
